# Role of hydrogen sulfide in the male reproductive system

**DOI:** 10.3389/fendo.2024.1377090

**Published:** 2024-05-30

**Authors:** Yunjia Song, Caiyun Mao, Qing Zhong, Rong Zhang, Deyou Jiang, Xutao Sun

**Affiliations:** ^1^ Department of Pharmacology, School of Basic Medical Sciences, Heilongjiang University of Chinese Medicine, Harbin, China; ^2^ Department of Typhoid, School of Basic Medical Sciences, Heilongjiang University of Chinese Medicine, Harbin, China; ^3^ Department of Synopsis of the Golden Chamber, School of Basic Medical Sciences, Heilongjiang University of Chinese Medicine, Harbin, China

**Keywords:** erectile dysfunction, H2S, testis, prostate cancer, oxidative stress, bladder

## Abstract

As an important gas signaling molecule, hydrogen sulfide (H_2_S) affects multiple organ systems, including the nervous, cardiovascular, digestive, and genitourinary, reproductive systems. In particular, H_2_S not only regulates female reproductive function but also holds great promise in the treatment of male reproductive diseases and disorders, such as erectile dysfunction, prostate cancer, varicocele, and infertility. In this review, we summarize the relationship between H_2_S and male reproductive organs, including the penis, testis, prostate, vas deferens, and epididymis. As lower urinary tract symptoms have a significant impact on penile erection disorders, we also address the potential ameliorative effects of H_2_S in erectile dysfunction resulting from bladder disease. Additionally, we discuss the regulatory role of H_2_S in cavernous smooth muscle relaxation, which involves the NO/cGMP pathway, the RhoA/Rho-kinase pathway, and K^+^ channel activation. Recently, various compounds that can alleviate erectile dysfunction have been reported to be at least partly dependent on H_2_S. Therefore, understanding the role of H_2_S in the male reproductive system may help develop novel strategies for the clinical treatment of male reproductive system diseases.

## 1 Introduction

In recent years, the incidence of male reproductive system diseases has increased, attracting substantial attention from researchers worldwide. Male reproductive system diseases primarily affect the penis, testis, prostate, vas deferens, and epididymis (1). Various microorganisms, environmental factors, and long-term smoking or alcohol abuse may affect male reproductive function (2–5), leading to diseases such as erectile dysfunction (ED) (6), prostate cancer (PCa) (7), varicocele (8), and infertility (9). Studies have shown that oxidative stress is an important factor contributing to the occurrence and development of these diseases. However, owing to the complex pathogenesis of male reproductive system diseases, the corresponding treatment strategies are not well-established. Therefore, identifying precise targets is necessary to optimize the diagnosis and treatment of male reproductive system diseases.

H_2_S is the third major gas signaling molecule after nitric oxide (NO) and carbon monoxide (CO) and possesses strong antioxidant activity (10). It is involved in the regulation of important pathophysiological processes, including inflammation, oxidative stress, autophagy, and apoptosis, in the cardiovascular, nervous, and digestive systems (11, 12). In addition, recent studies have shown that H_2_S affects the reproductive system in both men and women. H_2_S regulates female reproductive function through K^+^ channels and various signaling pathways, including the ERK1/2/NF-κB and Nrf2 pathways (13), and exerts protective effects on the male reproductive system. It plays an important role in early spermatogenesis and late maturation of spermatogenic cells and may prevent damage to the reproductive system by promoting the proliferation of spermatogonia, regulating the corpus cavernosum (CC) of the penis, and mediating erection as well as other related functions (14–16). In this review, we summarize the relationship between H_2_S and the male reproductive system, discuss the pathological mechanisms of male reproductive system diseases, and propose novel strategies for the early diagnosis (17) and prompt treatment of these diseases.

## 2 Distribution of H_2_S synthases in the male reproductive system

**Figure 1:**
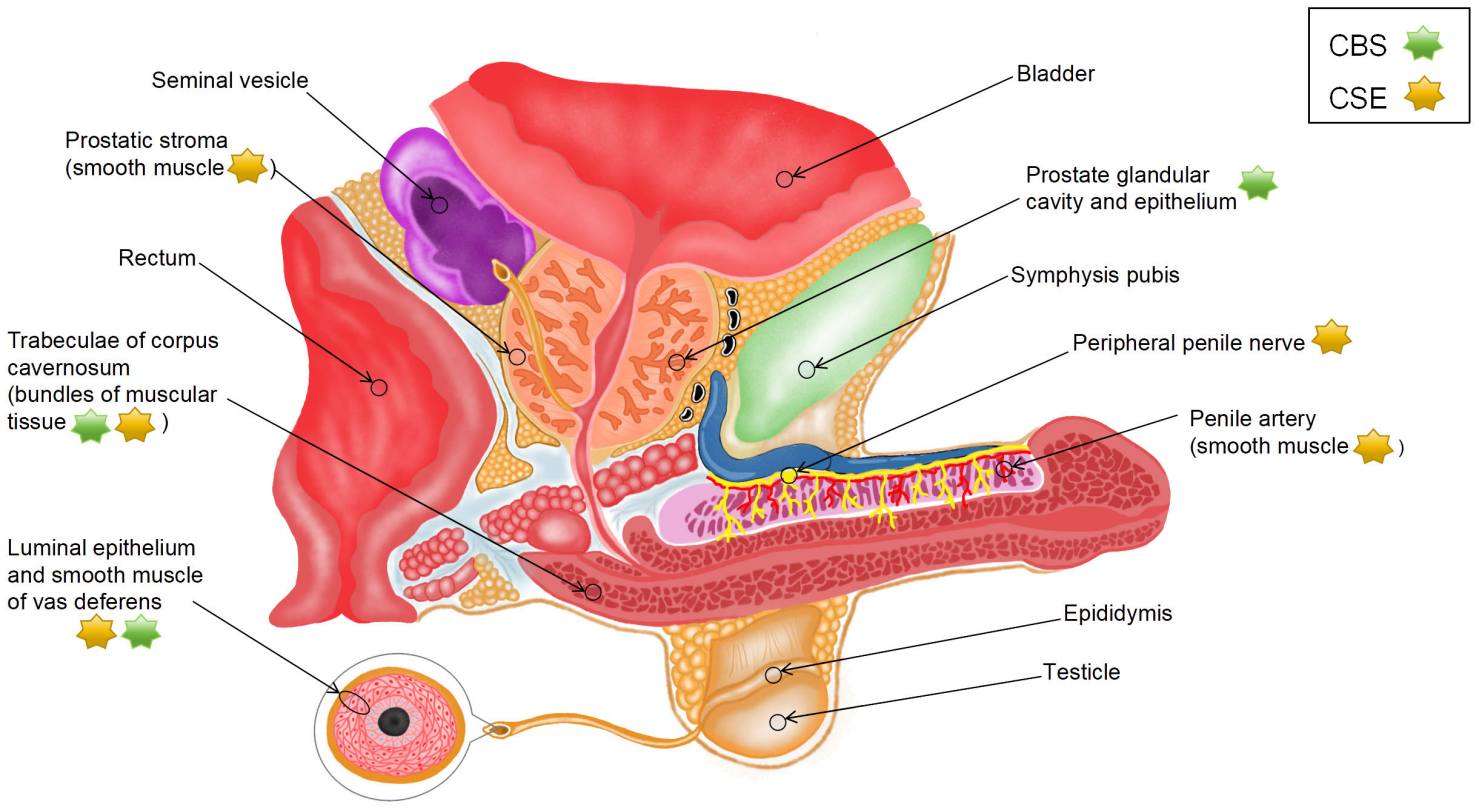
Distribution of H_2_S synthases in the male reproductive system.

Endogenous H_2_S is produced from L-cysteine (L-Cys) via desulfurization catalyzed by cystathionine γ-lyase (CSE) and cystathionine β-synthase (CBS) or from 3-mercaptopyruvate via 3-mercaptopyruvate sulfurtransferase (3-MPST) (18, 19). 3-mercaptopyruvate is derived from two sources as follows: L-Cys via cysteine aminotransferase (CAT) (CAT/3-MPST pathway) and D-cysteine via amino acid oxidase (DAO) (DAO/3-MPST pathway) (20). A study reported that H_2_S exhibited specificity to vascular smooth muscle (21), which may be related to differences in the mechanisms of H_2_S production. Given that endogenous H_2_S is synthesized through various pathways, the distribution of H_2_S synthases in various parts of male reproductive organs (**Figure 1**) may indicate the source and production mode of H_2_S.

Srilatha et al. (22) detected the presence of endogenous H_2_S in smooth muscle tissue homogenates from the CC of rabbits. Although they used rabbit CC and did not identify the exact source of H_2_S, their study was the first to show that H_2_S can be produced in the penis. Subsequently, d’Emmanuele et al. (23) evaluated the mRNA and protein expression of CBS and CSE in human corpus cavernosum (HCC) and validated that endogenous H_2_S was produced in HCC by the two enzymes. Immunohistochemical analysis showed that CSE was located in vascular smooth muscle cells (SMCs) in the penile artery, bundles of muscular tissue in HCC trabeculae, and peripheral nerves, whereas CBS was primarily located in bundles of muscular tissue in trabeculae. Furthermore, CBS and CSE have been found in human prostate tissue sections. CBS is mainly expressed in the glandular lumen and epithelial cells of the prostate, whereas CSE is distributed in the SMCs of the periacinar stroma (24, 25). These experimental results suggest that H_2_S is predominantly synthesized by CSE instead of CBS in male reproductive organs.

The tissue samples used in most existing studies have been derived from animals, such as rats and mice. However, the species and location of H_2_S synthases in animals are different from those in humans. In rats, all five H_2_S synthases, namely, CSE, CBS, CAT, DAO, and 3-MPST, are expressed in penile tissues at both mRNA and protein levels; however, CBS is not expressed in corpus cavernosum smooth muscle cells (CCSMCs) (26, 27). The mRNA expression of the five enzymes has been detected at two specific sites in rat prostate tissues (dorsolateral [PR-D] and ventral [PR-V]); however, these sites lack the protein expression of CSE and DAO. In particular, the expression of CBS is substantially higher in PR-V than in PR-D, the expression of 3-MPST is higher in PR-D than in PR-V, and the expression of CAT is similar between the two sites. Additionally, immunoreactivity of CBS, 3-MPST, and CAT has been observed primarily in rat glandular epithelial cells (19). In mice, CBS, CSE, and 3-MPST are primarily expressed in the CC (28). CSE is located in the endothelial cells of the cavern and vessels in the CC, with its expression being higher than that of CBS and 3-MPST. CBS is located in the cavernous subendothelial smooth muscle, vascular smooth muscle, and peripheral nerves, whereas CSE and 3-MPST are not found at these sites (29). A study showed that the expression of CSE in mouse prostate tissues substantially decreased with age, whereas that of CBS remained unaffected. Moreover, knockout of CSE reduced the production of H_2_S by approximately 80%, indicating that H_2_S is predominantly synthesized by CSE in the mouse prostate (30).

To the best of our knowledge, studies investigating the effects of H_2_S on the testis have used only experimental animal models; therefore, related data on human tissues are not yet available. In rats, CBS is abundant in Leydig and Sertoli cells, which can be observed in the interstitial space and basement membrane of seminiferous tubules, respectively. In addition, its expression is moderate in the immature reproductive cells of the peripheral region of the tubules but very low in the mature reproductive cells of the central region of the tubules. CSE is found in vascular walls in the interstitial space of the testis, Sertoli cells, and immature reproductive cells (31). CBS, CSE, and 3-MPST are expressed in the testis and germ cells in mice (32). Data on the vas deferens (VD) are scarce. A study on humans, rats, and mice showed that the expression of CSE and CBS was high in the luminal epithelium and smooth muscle of VD (33). Furthermore, the mRNA expression of CBS and CSE is high in the body and tail regions of rat epididymis (34). In particular, CBS is found in epididymal epithelial cells, whereas CSE is found in the thin layer of SMCs underneath the epididymal epithelium. These findings suggest that species-specific differences should be considered when using animals to investigate the effects of H_2_S on the reproductive system.

## 3 Effects of H_2_S on the male reproductive system

### 3.1 Effects of H_2_S on the penis

#### 3.1.1 Regulation of mechanisms underlying penile erection

Erection is a neurovascular event whose form and function mostly depend on the relaxation of the cavernous smooth muscle and arteries in the penis. When parasympathetic nerves are stimulated to release neurotransmitters, the smooth muscle of HCC (a highly vascularized structure) and human penile resistance arteries relax. Subsequently, blood flows into the cavernous sinuses and inflates them, which in turn pressurizes the veins to reduce blood outflow and increases intracavernosal pressure (ICP), eventually leading to penile erection (35). Altogether, arterial blood flow exceeding venous blood return causes and maintains normal penile erection, with smooth muscle relaxation playing a key role in the process.

The neurotransmitter-like regulatory effects of H_2_S on vascular smooth muscle have been reported in previous studies (36). Srilatha et al. (37) were the first to demonstrate that H_2_S has a neuroregulatory effect on penile erection. They examined the effects of exogenous H_2_S on the penis by injecting sodium hydrosulfide (NaHS) into the CC of non-human primates. The results showed that both penile length and ICP were remarkably increased, and the changes before erection were similar to those observed in the positive control group (injected with prostaglandin E1). Furthermore, injection of DL-propargyl glycine (PAG, a CSE inhibitor) into the external jugular vein of rats notably weakened the ICP induced by electrical stimulation of the cavernous nerve with platinum wire electrodes. These findings suggest that neuronal excitation induces the release of H_2_S to mediate penile erection. The detection of CSE in the human peripheral penile nerve supports these findings (23). In addition, Jupiter et al. (38) demonstrated that exogenous H_2_S promoted penile erection in rats.

At present, the exact mechanism through which H_2_S relaxes the cavernous smooth muscle remains unclear. The previously reported mechanisms include synergistic action of H_2_S with NO, activation of K^+^ channels, and regulation of the RhoA/ROCK pathway (**Table 1**); among which, the first mechanism is the most controversial. NO is considered the primary mediator of erectile function (56). Endogenous NO is generated by NO synthase (NOS) from L-arginine and consists of three isoforms as follows: neuronal NOS (nNOS), endothelial NOS (eNOS), and inducible NOS (iNOS) (57). NO increases cGMP levels by activating soluble guanylate cyclase (sGC); subsequently, cGMP acts as a second intracellular messenger to regulate calcium channels and contractile proteins involved in the relaxation of cavernous smooth muscle (58). As early as 1997, Hosoki et al. (59) proposed the possibility of synergism between H_2_S and NO. They found that 100-μM NaHS had a weak effect on relaxing thoracic aortic smooth muscle in rats; however, when administered in the presence of 10-nM sodium nitroprusside (SNP, an NO donor), NaHS strongly relaxed the smooth muscle. Similarly, in the presence of 30-μM NaHS, SNP increased the relaxation of thoracic aortic smooth muscle by 13 times.

**Table 1:** Effects of H_2_S on the male reproductive system.

Organ	Action	Mechanisms	Models	References
Penis	Promotion of erection	Activation of the RhoA/ROCK pathway and K_ATP_ channel	HCC strips from transsexual operation (n = 6)	(23)
		Activation of the sGC/cGMP pathway	CC strips from men with ED (n = 50)	(39)
		Activation of the NO/sGC/cGMP pathway	CSE^-/-^ mice	(16)
		Activation of the RhoA/ROCK pathway	Mouse CC strips	(40)
		Dependent on cAMP or cGMP	Rabbit CC strips (n = 5)	(22)
		Activation of the BKCa channel	Anesthetized rats	(38)
		Activation of BKCa and Kv channels	Rat CC strips	(41)
		Increased eNOS expression	L-NAME-induced hypertensive rats (n = 40)	(42)
		Increased NO levels and HO activity	Diabetic rats (n = 90)	(43)
		Activation of the RhoA/ROCK pathway	Rats with bilateral cavernous nerve injury (n = 18)	(44)
		Activation of the NO/sGC/cGMP pathway and K_ATP_ channel	Rats with STZ-induced diabetes (n = 10 or 12)	(45)
	Improve vascular injury of CC	Inhibition of the TGF-β1/Smad/CTGF pathway	Rats with STZ-induced diabetes	(46)
Prostate	Inhibition of CRPC	S-sulfhydration of AR	CSE knockout and overexpression in LNCaP cells and LNCaP-B cells	(30)
	Promotion of NE differentiation	Increased activity of Cav3.2	LNCaP cells	(47)
Testis	Reduce sperm motility	Activation of AMPK/Akt-related pathways	Boar sperm; NH_4_Cl- and/or Na_2_S-treated mice	(48)
	Alleviate the apoptosis of testicular germ cells	Increased SOD activity and reduced Bax/Bcl-2 ratio	Mice subjected to heat exposure	(32)
		Reduction of iNOS, TNF-α, and Apaf-1 levels	Rats with testicular torsion-induced I/R injury (n = 38)	(49)
		Activation of the Keap1/Nrf2 signaling pathway	GC-2spd(ts) cells derived from mouse spermatocytes	(50)
	Alleviate testosterone synthesis	S-sulfhydration of PDE4A/8A and activation of the cAMP/PKA pathway	Mouse Leydig tumour cells with LPS + H2O2-induced testosterone synthesis impairment	(51)
	Increase sperm motility	Activation of the CBS/H_2_S pathway	Mice with LPS- and diabetes-induced sperm dysfunction and CBS^+/‐^ mice	(52)
		ROS scavenging	Fe^2+^/ascorbate-treated boar sperm	(53)
Epididymis	Alleviate varicocele-induced epididymis injury	Activation of the PI3K/Akt pathway	Experimental varicocele rat model (n = 60)	(54)
	Maintain quiescence of epididymal sperm	Activation of K_ATP_ and BKCa channels	Cauda epididymal epithelium cells	(34)
Vas deferens	Regulation of VD spontaneous contraction	Activation of the L-Cys/H_2_S pathway	Human VDs from monorchidism surgery (n = 3); rat VDs (n = 20); mouse VDs (n = 11)	(33)
		Activation of BKCa channel	Rat VDs	(55)

Akt, protein kinase B; AMPK, adenosine 5’-monophosphate AMP-activated protein kinase; Apaf-1, apoptosis protease-activating factor-1; BKCa, large-conductance Ca^2+^-activated K^+^ channel; CRPC, castration-resistant prostate cancer; cAMP, cyclic adenosine monophosphate; cGMP, cyclic guanosine monophosphate; CTGF, connective tissue growth factor; CC, corpus cavernosum; ED, erectile dysfunction; NOS, NO synthase; ERK1/2, extracellular signal-regulated kinase ½; HCC, human corpus cavernosum; HO, heme oxygenase; Keap1, Kelch-like ECH-associated protein 1; L-NAME, nω-nitro-L-arginine; L-Cys, L-cysteine; LPS, lipopolysaccharide; NE, neuroendocrine; PCa, prostate cancer; Nrf2, nuclear factor erythroid 2-related factor 2; PI3K, phosphatidylinositol 3’-OH kinase; ROCK, Rho-kinase; sGC, soluble guanylate cyclase; SOD, superoxide dismutase; STZ, streptozotocin; VD, vas deferens.

On the contrary, the results of some studies on CC do not support the synergism between NO and H_2_S. Srilatha et al. (22) incubated noradrenaline-precontracted rabbit cavernosum tissue strips with n^ω^-nitro-L-arginine (L-NAME, a non-selective NO synthase inhibitor) and subsequently treated them with NaHS (100 μM–3.2 mM). The results showed that muscle relaxation induced by NaHS was not affected by L-NAME. Furthermore, the strips were precontracted in the presence of guanethidine and atropine and incubated with aminooxyacetic acid (AOAA, a CBS inhibitor) and β-cyanoalanine (β-CA, a CSE inhibitor) or PAG, respectively. After electrical stimulation, H_2_S inhibitors did not affect non-adrenergic, non-cholinergic nitrergic (NANC) relaxation. Similarly, L-NAME had minimal inhibitory effects on muscle relaxation induced by NaHS (1 μM–10 mM) (23). A study on live rats showed that intracavernosal injection of sodium sulfide (Na_2_S, 0.03–1 mg/kg) increased ICP, whereas intravenous injection of L-NAME had no considerable effect on ICP (38). Moreover, intracavernosal injection of Na_2_S did not alter the SNP-induced erectile response. In mouse CC, NO deficiency may increase the expression of CSE and 3-MPST, leading to an increase in the production of H_2_S and H_2_S-induced muscle relaxation (28). This effect may be compensatory and disproves that the relaxation effects of H_2_S are dependent on NO. Altogether, the abovementioned findings indicate that H_2_S does not promote penile erection through synergism with NO.

Some studies investigating the relationship between H_2_S and NO in the CC tissue support the synergy between them. H_2_S has been shown to increase the expression of NOS. Meng et al. (39) showed that the mRNA and protein levels of eNOS were remarkably higher in rat CC tissues treated with NaHS than in untreated tissues, whereas those of nNOS were not considerably different between treated and untreated tissues. Yilmaz et al. (42) showed that L-NAME decreased the protein expression of eNOS and nNOS in the penile tissues of hypertensive rats, whereas addition of NaHS (0.037 mg/kg) prevented this change. Consistent with these two studies, another study showed that NaHS (30 mg/kg) increased NO levels in rat CC tissues (43). Furthermore, H_2_S has been shown to increase the activity of eNOS. Meng et al. (39) showed that eNOS notably increased the production of NO from L-arginine by 5 times in more than rat CC tissues treated with NaHS (1 mM), suggesting that H_2_S enhanced the activity of eNOS. Knockout of CSE may not alter total eNOS levels but may remarkably reduce the levels of its active form p-eNOS (16).

These contradictory findings suggest that tissue- and species-specific crosstalk exists between H_2_S and NO and the mechanism through which H_2_S relaxes the cavernous smooth muscle is independent of the NO/cGMP signaling pathway. Endogenous H_2_S can inhibit the targeted degradation of cGMP by inhibiting phosphodiesterase (PDE) (60). Several studies have demonstrated that H_2_S in the penis regulates cGMP levels by acting on sGC. Stimulation of HCC with both endogenous and exogenous H_2_S (L-Cys [1 μM–1 mM] and NaHS [1 μM–1 mM], respectively) increases cGMP levels, which may be restored upon treatment with 1H-[1,2,4]oxadiazolo[4,3-a] quinoxalin-1-one (ODQ, an sGC inhibitor) (61). The reduction of H_2_S levels in CSE-knockout mice results in an impaired redox state of sGC, decreasing cGMP levels in the penis (16). These findings suggest that H_2_S bypasses NO to upregulate cGMP and hence affects its downstream signaling.

The four primary types of potassium (K^+^) channels expressed in arterial smooth muscle cells include Ca^2+^-activated (KCa), adenosine triphosphate (ATP)-sensitive (K_ATP_), inwardly rectifying (Kir), and voltage-gated (Kv) channels (62). The relaxation effects of H_2_S on vascular SMCs isolated from rats have been shown to rely on the K_ATP_ channel (21). The four K^+^ channels have also been detected in HCC (63). To examine the role of these K^+^ channels in H_2_S-mediated responses in CC, Jupiter et al. (38) injected tetraethylammonium (TEA, a non-selective K^+^ channel inhibitor), iberiotoxin (a large-conductance Ca^2+^-activated K^+^ [BKCa] channel inhibitor), and glybenclamide (GLB, a K_ATP_ channel inhibitor) into the CC of anesthetized rats and examined their effects on Na_2_S (0.03–1 mg/kg)-induced changes in ICP. The results showed that intracavernosal injection of Na_2_S induced an increase in ICP, which was attenuated by TEA and iberiotoxin but not by GLB. In an organ bath experiment, Abd Elmoneim et al. (41) treated rat CC tissues with TEA, GLB, 4-aminopyridine (4-AP, a Kv channel inhibitor), and barium chloride (BaCl2, a Kir channel inhibitor) to examine their effects on relaxation induced by L-Cys (1 μM–10 mM). The results showed that TEA and 4-AP remarkably attenuated relaxation, with the effects of TEA being stronger than those of 4-AP, whereas GLB and BaCl2 failed to reduce relaxation. These findings indicate that BKCa and Kv channels are involved in the H_2_S-induced relaxation of rat CC, whereas Kir and K_ATP_ channels may not participate in the process. In another study, GLB was found to attenuate the relaxation effects of NaHS on HCC strips (23); however, the dose of GLB used was 150 μM. When administered at a dose of >10 μM, GLB inhibits the Na^+^–K^+^ pump and L-type Ca^2+^ channel (64), which may interfere with the results. Consistently, studies have shown that treatment with GLB (10 μM) and NaHS does not suppress relaxation in rat (26, 41) or human (61) cavernosal tissue strips.

RhoA is a monomeric GTP enzyme that is activated upon binding to GTP, subsequently stimulating ROCK (a serine/threonine kinase) (65). ROCK phosphorylates the myosin-binding subunit of myosin light chain (MLC) phosphatase, which is responsible for the dephosphorylation of MLC, to inactivate the enzyme (66). In addition, it directly phosphorylates MLC, causing myosin to bind to β-actin, which promotes smooth muscle contraction (67). Chitaley et al. (68) used (*R*)-(+)-*trans*-N-(4-pyridyl)-4-(1-aminoethyl)-cyclohexanecarboxamide (Y-27632, a specific ROCK inhibitor) to examine the effects of ROCK on the cavernosal tone in rats. They found that inhibition of ROCK induced an increase in ICP, which stimulated penile erection in rats. This effect was found to be independent of the NO pathway. d’Emmanuele et al. (23) found that the relaxation effects of NaHS (1 μM–10 mM) were considerably stronger in HCC strips precontracted with U46619 or human endothelin-1 (two ROCK pathway modulators) that in those precontracted with phenylephrine (PE). This finding indicates that H_2_S regulates the ROCK pathway. Consistently, another study showed that fasudil (a ROCK inhibitor) reduced the relaxation response of mouse CC to exogenous H_2_S, suggesting that an interaction between H_2_S and ROCK is highly likely (69). Aydinoglu et al. (40) were the first to report that ROCK participates in the relaxation-inducing effects of H_2_S in mouse CC contracted with PE. They found that pre-treatment with Y-27632 remarkably reduced cavernosal muscle contraction induced by the PE-driven phosphorylation of the myosin phosphatase-targeting subunit 1 (MYPT1) at Thr696. However, PE-induced muscle contraction almost disappeared in the presence of L-Cys or NaHS. Correspondingly, the combination of Y-27632 and L-Cys (10 mM) or NaHS (1 mM) inhibited the expression of phosphorylated MYPT1, whereas PAG and AOAA reversed this change. Furthermore, Y-27632 increased the basal and L-Cys-induced production of H_2_S, which was attenuated by PAG and AOAA. These findings indicate that ROCK at least partly inhibits CSE/CBS in CCSMCs. In addition to regulating the phosphorylation of MLC, the RhoA/ROCK signaling pathway affects the phenotypic modulation of CCSMCs by regulating the downstream factors CDK2, Cyclin E_1_, and P27^kip1^, thereby promoting smooth muscle contraction (44). A study showed that NaHS (100 μmol/kg) inhibited the phenotypic transformation of CCSMCs induced by the upregulation of RhoA/ROCK signaling, thereby improving erectile function in rat models of bilateral cavernous nerve injury (BCNI) (44).

In addition to playing an important role in the abovementioned contraction mechanisms, H_2_S may be involved in other less investigated pathways of CC relaxation, such as the CO/heme oxygenase-1 (HO-1) pathway (43). Notably, the H_2_S donor used in a majority of the abovementioned studies is NaHS, whose H_2_S release rate may not be sufficient to accurately mimic endogenous H_2_S production. As a slow-H_2_S-releasing donor, GYY4137 is more suitable for investigating the effects of H_2_S on physiological and pathological processes. Qabazard et al. (45) found that the effects of GYY4137 on the relaxation of rat CC were at least partly mediated by the NO pathway and K_ATP_ channel. However, only a few studies have reported the use of GYY4137 to treat penile tissues. Moreover, GYY4137 produces CO, a by-product that acts in a similar way to H_2_S (70). Therefore, more experimental data are required to support the conclusion of existing studies. An in-depth understanding of the mechanisms through which H_2_S regulates penile erection may guide the development of novel therapeutic approaches for ED.

#### 3.1.2 Therapeutic targets and future perspectives to treat ED

Penile erection involves the cooperation of nerves, blood vessels, and smooth muscle; consequently, lesions or damage in any part of these nerves, blood vessels, and smooth muscle may lead to ED. Neurovascular damage, diabetes, hypertension, side effects of drugs, and testosterone deficiency have been identified as causes of ED (71). PDE-5 inhibitors (PDE-5is) (such as sildenafil and tadalafil) are considered the first-line treatment for ED (72); however, some patients have poor outcomes. Before the erection-promoting effects of H_2_S were reported, a novel target, β3-adrenergic receptor, was identified. β3-adrenergic receptors are present in human CCSMCs and cause smooth muscle relaxation in HCC in a cGMP-dependent but NO-independent manner upon activation (73). Mitidieri et al. (74) found that activation of β3 receptors by BRL37344 (a β3-selective agonist) relaxed HCC and penile arterial rings in an H_2_S/cGMP-dependent manner, whereas inhibition of CSE notably reduced the relaxation. Treatment with BRL37344 considerably increased H_2_S production, whereas inhibition of CSE reduced the BRL37344-induced increase in cGMP expression in both tissues. Given that their function is independent of the endothelium, selective β3 agonists, such as mirabegron (75), may serve as alternative treatment agents for patients who do not respond to PDE-5is.

ED has been associated with defects in the L-Cys/H_2_S pathway (76). The levels of H_2_S synthases are decreased to varying degrees in the penile tissues of rats with ED caused by radical prostatectomy, hyperlipidemia, diabetes, or hypertension (27, 42, 44, 77). This phenomenon suggests that ED can be treated with exogenous H_2_S supplementation. The first drug developed was H_2_S-donating derivative of sildenafil (ACS6) (78). Although the muscle relaxation effects of ACS6 are similar to those of sildenafil citrate at the same concentration, ACS6 is more effective than sildenafil and NaHS in reducing superoxide formation and PDE5 expression. Theoretically, long-term use of ACS6 may improve ED by inhibiting oxidative stress and downregulating PDE5. Several natural plant extracts have been found to stimulate H_2_S synthesis *in vivo*. For example, resveratrol (RVT) (79), found in red wine, causes CC relaxation in a concentration-dependent manner in mice. This effect can be reversed by CBS inhibitors but not by L-NAME. Although RVT increases the basal and L-Cys-induced production of H_2_S, it does not affect NaHS-induced relaxation. These results suggest that RVT-induced relaxation is at least partly dependent on H_2_S production, does not interfere with the downstream mechanisms of H_2_S production, and is independent of NO. Sodium tanshinone IIA sulfonate (STS) (77), a water-soluble derivative of lipophilic diterpene isolated from the roots of Danshen plants, can reverse the high fat diet-induced decrease in CBS and CSE expression and H_2_S production in rats. In addition, it can preserve erectile function by activating the Nrf2/HO-1 pathway against high fat diet-induced oxidative stress.

Because H_2_S is primarily released by SMCs instead of endothelial cells, it may serve as a promising therapeutic target in patients with ED with endothelial dysfunction, such as those with metabolic syndrome and diabetes. A study showed that high-fructose diet-induced metabolic syndrome led to a reduction in the basal and L-Cys-induced production of H_2_S in rat penile tissues (80), whereas supplementation with exogenous H_2_S improved erectile function. GYY4137, an H_2_S donor with sustained release, has been shown to improve cavernosal vascular reactivity by inhibiting the TGF-β1/Smad/CTGF pathway in rats with STZ-induced diabetes (46). In addition, long-term treatment with GYY4137 can prevent or attenuate the reduction of H_2_S levels and improve cavernosal H_2_S production in diabetes (45, 46). The combination of H_2_S donors and PDE-5is holds great promise in the treatment of ED. A study showed that NaHS combined with tadalafil was more effective than NaHS alone in the treatment of ED in rats with partial bladder outlet obstruction (81). The reduced erectile response and H_2_S levels were only partially restored upon treatment with NaHS but completely restored upon treatment with both NaHS and tadalafil. In addition, the combined use of NaHS and tadalafil reversed the morphological and functional changes in the penis caused by ischemia after obstruction and had a positive effect on the erectile response. These results suggest that H_2_S can improve ED and restore spontaneous erection with long-term use.

### 3.2 Effects of H_2_S on the prostate

PCa, and particularly castration-resistant prostate cancer (CRPC), is the primary focus of research on H_2_S’ utilities on the prostate. Because the growing and progression of the prostate depend on androgens, androgen deprivation therapy is the mainstay of treatment for advanced PCa. However, most patients inevitably progress to androgen-independent castration resistance, which is a leading cause of death in patients with PCa (82). Given that the signals generated upon the biding of the androgen receptor (AR) to testosterone or 5α-dihydrotestosterone are closely related to the progression of PCa to CRPC, direct inhibition of AR is one of the widely used therapeutic strategies for PCa (82, 83). The highly conserved DNA-binding domain, one of the four domains of AR, contains two cysteine type 4 zinc fingers; of which, the second zinc finger is the binding site for homodimerization (83). According to Zhao et al. (30), H_2_S suppresses transactivation of AR by S-sulfhydrating cysteine Cys611 and Cys614 sites of its second zinc finger, thereby inhibiting the progression of antiandrogen-resistant PCa cells. Bicalutamide competes with AR for binding, making it an effective cancer treatment. It has been reported that PCa (LNCAP-B) cells resistant to bicalutamide expressed less of CSE than PCa (LNCaP) cells dependent on androgens. LNCaP-B cells overexpressing CSE or administered NaHS re-established sensitivity to bicalutamide, while CSE-deficient LNCaP cells persist in growing with the utilization of bicalutamide. On the contrary, changes in CBS expression under the same conditions did not have obvious effects on drug resistance. Therefore, it is possible to use the CSE/H_2_S system to assess prognosis and to treat early PCa and CRPC. However, Fukami et al. (47) showed that the effects of H_2_S were not beneficial to the treatment of PCa. They showed that androgen deprivation-induced enhancement of cytosolic cAMP elevated CSE expression and H_2_S production. A subsequent study found that H_2_S enhanced the activity of Cav3.2, which led to an increase in proliferation of tumors independent of androgen. Neuroendocrine phenotypes account for approximately 20–25% of all metastatic CRPC cases. In most cases, neuroendocrine differentiation is induced by androgen deprivation therapy (84). Differentiation of LNCaP cells is characterized by the upregulation of Ca^2+^-dependent secretion of mitogenic factors and the overexpression of Ca_v_3.2 T-type Ca^2+^ channels that contribute to their secretion (85, 86). Fukami et al. showed that differentiated cells had increased expression of CSE and CBS and elevated T-type Ca^2+^ channel-dependent currents (T-currents). The T-currents were suppressed by CSE inhibitors but not by CBS inhibitors and were enhanced by H_2_S donors (NaHS, 0.1–1.5 mM; Na_2_S, 0.03–0.1 mM). These results indicate that in LNCaP cells undergoing neuroendocrine differentiation, H_2_S is able to stimulate T-type Ca^2+^ channels, leading to the development of neuroendocrine CRPC.

The abovementioned studies indicate that CSE/H_2_S is associated with the progression of PCa, and the contradictory conclusions could be attributed to the fact that PCa cells respond differently to H_2_S depending on their source, and dose and type of H_2_S donor (87). Research shows that drugs related to H_2_S are effective in treating cancer. Multi-cancer cell lines, including PCa cells, are inhibited 28–3000 times more effectively by H_2_S-releasing non-steroidal anti-inflammatory drugs (HS-NSAIDs) than by those of conventional NSAIDs (88). HS-ibuprofen is 200 times more potent than ibuprofen in LNCaP cells owing to its covalent attachment to the H_2_S-releasing moiety. The H_2_S moiety of H_2_S-releasing doxorubicin (H_2_SDox) exerts cardioprotective effects, reducing the cardiovascular side effects of doxorubicin (89). By releasing the SH2 group, disulfide bonds formation on P-gp would disrupt P-gp activity, ultimately improving tumor sensitivity to doxorubicin. A study showed that intracellular drug accumulation was substantially higher after H_2_SDox treatment than after doxorubicin treatment in DU-145 PCa cells resistant to androgen and doxorubicin. Zhou et al. (90) combined H_2_S with classical drugs and used nanotechnology to develop Zn^2+^-interference and H_2_S-induced therapeutics, which responds to the tumor microenvironment (TME) and is derived from tannic acid (TA)-altered zinc sulfide nanoparticles (ZnS@TA). ZnS@TA nanoparticles responded specifically to tumor cells based on the pH. H_2_S and Zn^2+^ were released in a small amount in a neutral environment (pH = 7.4) but had good degradation performance in a simulated TME. Consistently, ZnS@TA nanoparticles had no obvious effects on the viability of DU-145 cells under neutral conditions but considerably decreased cell viability in the TME at the same concentration. ZnS@TA nanoparticles attenuated the migratory and invasive abilities of PCa cells by increasing intracellular TA and Zn^2+^ levels. Subsequently, Zn^2+^ ions and H_2_S synergistically enhanced tumor cell apoptosis.

### 3.3 Effects of H_2_S on the testis

**Figure 2:**
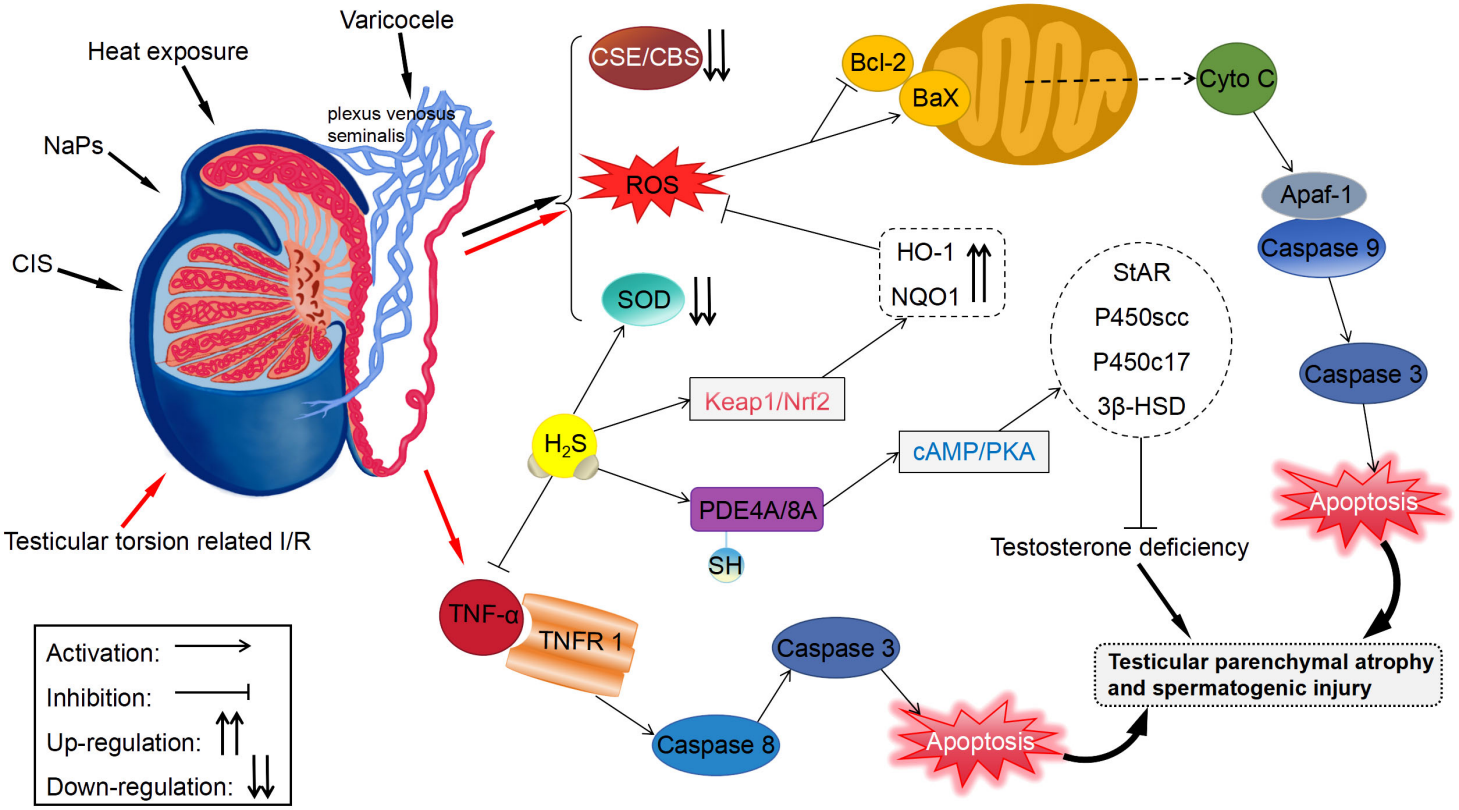
Protective effects of H_2_S on the testis and germ cells. Apaf-1, apoptosis protease-activating factor-1; HO-1, heme oxygenase-1; NQO1, NADPH dehydrogenase quinone 1.

As a gaseous air pollutant, H_2_S may impair spermatogenesis (91) and inhibit sperm mobility via mechanisms related to AMPK/Akt (48) when combined with NH_3_, thereby disrupting male fertility. However, there are several investigations indicate that H_2_S can protect the testis and germ cells by inhibiting inflammation, oxidant activity, and apoptosis (**Figure 2**). The testis and sperm are highly susceptible to oxidative stress-induced damage. Studies have shown that physical stimulation (heat stress and restraint stress) (92), ischemia–reperfusion (I/R) injury, varicocele (testicular torsion), and ingestion of reproductive-toxic substances (cisplatin and nanoplastics) can increase the production of reactive oxygen species (ROS) in the testis, leading to testicular dysfunction and germ cell apoptosis. In addition, the aforementioned conditions may decrease the expression of CBS and CSE and production of H_2_S in the testis. Therefore, low levels of H_2_S in the testis may play a key role in male infertility.

Li et al. (32) stimulated the testis of mice with heat exposure (42°C, 30 minutes/day, 3 days) and found that heat stress remarkably elevated ROS generation and inhibited the SOD activity in germ cells. However, exogenous administration of NaHS stimulated SOD activity and reduced ROS generation. Mechanistically, H_2_S can inhibit cytochrome C release and Bax/Bcl-2 ratio, thus impeding the heat stress-induced testicular germ cells apoptosis. Bozkurt et al. (49) found that H_2_S alleviated excessive tissue detriment by reducing the levels of iNOS and the inflammatory cytokine TNF-α in a rat model of testicular torsion-induced I/R damage. Furthermore, H_2_S suppressed apoptosis through reducing apoptosis protease activating factor-1 level, thus protecting against testicular damage. Patients with varicocele are usually treated with surgery; however, the removal of the varicose veins (varicocelectomy) carries not only the risk of surgery but also the risk of postoperative side effects, including recurrence, hydrocele formation, atrophy, and bleeding (93). Rats with left varicocele exhibited significant reductions in left testicle and epididymis weights, as well as diameters and epithelial thicknesses of the seminiferous tubules. When NaHS was administered over a long period, it reduced oxidative stress and apoptosis in the testicles, restoring above results (94). According to the study by Xia et al. (54), GYY4137 had beneficial effects on rats with varicocele-induced ipsilateral epididymis damage and sperm injury through stimulating PI3K/Akt signaling. Additionally, Shafie et al. (95) reported that testosterone combined with NaHS alleviated varicocele-induced injury in rats. It was shorter in duration and required lower doses than using NaHS for a long period of time when testosterone was combined with NaHS.

Cisplatin (CIS), an antineoplastic drug increases the levels of free radicals and decreases those of antioxidant enzymes or non-enzymes in testicular tissue via oxidative stress, resulting in testicular toxicity (96). It was observed in rats testicular tissues that administration with CIS notably elevated the content of MDA, a direct indicator of ROS-induced lipid peroxidation, and inhibited the activity of SOD (97). Whereas, CIS-induced changes in biochemistry, histology, and morphology could be effectively reversed with NaHS treatment. Ingested polystyrene nanoparticles can accumulate in the testis and cause testicular atrophy, degeneration of spermatogenic tubules, and spermatogenesis disorders (98, 99). Using mouse spermatocytes, Li et al. (50) observed that nanoplastics caused autophagy and apoptosis dependent on ROS, leading to reproductive noxiousness. Conversely, H_2_S mitigated nanoplastic-mediated reproductive noxiousness via upregulating antioxidant enzymes level, including HO-1 and NQO1, through the Keap1/Nrf2 pathway. Additionally, testosterone inadequacy may adversely impact sperm morphology and vitality, potentially compromising male fertility (100). A mouse Leydig tumor cell line with H_2_O_2_ + LPS-induced testosterone deficits was overexpressed of CBS to restore testosterone levels by S-sulfhydrylating PDE4A and PDE8A and stimulating the cAMP/PKA signaling (51). Another study showed that animal models of deficient H_2_S production had lowered sperm vitality, which was alleviated upon exogenously administering H_2_S or overexpressing CBS (52). The stress-induced decrease in endogenous H_2_S generation was involved in damaged spermatogenesis and a defective blood–testis barrier.

It is possible that H_2_S may enhance sperm vitality *in vitro*, which could have implications for assisted reproduction. During the extraction of human sperm from a sperm bank for artificial insemination, freezing and thawing can produce ROS, resulting in oxidative stress-induced impairment to sperm (101). H_2_S donors are capable of maintaining sperm vitality, reducing acrosomal deprivation, and protecting sperm against oxidative stress (53). In particular, a high concentration of H_2_S attenuates sperm movement (102). In contrast, the simultaneous administration of low concentrations of H_2_S and NO donors has been shown to promote sperm frontward movement and safeguard plasma membrane against oxidative stress (103). According to Pintus et al. (104), short-term treatment with two N-thiocarboxyanhydrides derived from glycine and leucine enhanced the mitochondrial activity of pig sperm cells even in the presence of ROS. The two amino acid-based H_2_S-releasing drugs can mimic the physiological H_2_S release when carbonic anhydrase is present without causing cellular harm. Additionally, they can enhance sperm vitality after short-term treatments, consequently prolonging sperm survival.

### 3.4 Effects of H_2_S on other male reproductive organs

Studies investigating the effects of H_2_S on the epididymis and VD are limited. The contraction of the epididymis and VD contributes to the discharge of sperm. Studies have shown that the L-Cys/H_2_S pathway is involved in the regulation of VD tonic contractions and that H_2_S relaxes VD smooth muscle in a concentration-dependent manner (33, 55). Based on these findings, the mechanisms underlying VD relaxation have been investigated in some studies. Li et al. (55) treated VD bands with 2-APB, an inhibitor of transient receptor potential [TRP] channels, and apamin, an inhibitor of Ca^2+^-activated K^+^ [SKCa] channels, as well as L-NAME, TEA, iberiotoxin, GLB, and subsequently treated them with NaHS. According to the results, L-NAME, GLB, 2-APB, and apamin had no influence on the relaxation of VD to NaHS, while TEA and iberiotoxin considerably reduced it. These findings indicate that H_2_S may target BKCa channels in VD. Moreover, N-ethylmaleimide safeguards thiols against oxidation by alkylation, counteracting NaHS-mediated smooth muscle relaxation in VD; however, the strong reducing agent DTT, which can disrupt disulfide bonds in proteins, did not alter the effect of NaHS. The results indicate that H_2_S may impact the function of BKCa channels in VD smooth muscle by S-sulfhydration, consequently leading to muscle relaxation. In the aforementioned study, NaHS was used at a concentration of 1 mM, which exceeds the physiological concentration of H_2_S. Therefore, whether H_2_S regulates spontaneous contractions in VD in the physiological state remains unknown. In the epididymal lumen, the microenvironment supports sperm vitality, and mature sperm are stocked in the epididymis’ tail until ejaculation (105). Gao et al. (34) found that H_2_S induced remarkable K^+^ release from the epididymal epithelium in rats through stimulating K_ATP_ and BKCa channels. An increase in K^+^ concentration in the cauda epididymal intraluminal fluid inhibited sperm vitality regardless of pH. Consequently, H_2_S created a microenvironment with an elevated K^+^ concentration in the cauda epididymis lumen, which maintained epididymal sperm quiescence prior to ejaculation. According to these findings, H_2_S plays a significant role in ejaculation. It is possible that future studies will lead to the development of novel strategies for treating asthenospermia, spermatorrhea, as well as premature ejaculation.

## 4 Effects of H_2_S on other organs in the genitourinary system

In addition to the aforementioned components of the male reproductive system, the kidneys, ureters, bladder, and urethra are also impacted by H_2_S. Among these, the kidneys have been the subject of extensive research, particularly in relation to acute kidney injury, chronic kidney disease, kidney cancer, and other related conditions. Despite this, the bladder is often the primary organ considered in discussions of the genitourinary system due to its close association with the reproductive system. Specifically, the correlation between ED and lower urinary tract symptoms (LUTS) has been a topic of widespread examination.

H_2_S has been shown to potentially alleviate LUTS and associated ED by inducing relaxation of the smooth muscle in the bladder. Male LUTS encompasses a range of structural, functional, and sensory abnormalities affecting the lower urinary tract, including pelvic and pelvic floor organs such as the bladder, prostate, and urethra (106). The presentation of LUTS is multifaceted, with symptoms categorized into storage, voiding, and post-voiding symptoms, often occurring concurrently in affected individuals (107). The correlation between ED and LUTS has been extensively studied in recent years, with a growing body of evidence indicating a strong link, particularly in men with LUTS who are at a higher risk of developing ED (108, 109). For instance, a comprehensive population-based study examining the impact of overactive bladder, specifically urgency as a storage symptom, on male sexual health revealed a significant association with ED (110).

The primary mechanism of action for current first-line medications used in the treatment of LUTS involves the relaxation of bladder smooth muscle (111). The process of bladder contraction during urination is primarily regulated by cholinergic, adrenergic, and sensorimotor nerves (112). Early research indicated that the effect of H_2_S on the bladder mirrors that of capsaicin. In rats, NaHS prompted bladder contractions by stimulating primary afferent nerve (sensory nerve) terminals to release tachykinin, which subsequently activated NK1 and NK2 receptors (113). It is noteworthy that sensory nerves rapidly developed desensitization to H_2_S, leading to the cessation of contractions. Additionally, the application of H_2_S to desensitized sensory nerves resulted in the release of inhibitory neuropeptides and induced bladder relaxation (114). As the research progressed, Fernandes et al. (115) determined that GYY4137 activates L-type Cav channels in a concentration-dependent manner to enhance ACh release from guinea pig bladder neurons, thereby increasing the amplitude of phasic contraction of bladder smooth muscle. These experiments utilized isolated bladder strips with the urothelium removed, despite the presence of H_2_S not only in the nerve fiber and smooth muscle layers of the bladder but also in the urothelium (19, 25, 116, 117). Consistently, activation of M1/M3 receptors in the human urothelium leads to the phosphorylation of CBS at Ser227 through the cGMP/PKG pathway, resulting in elevated epithelial H_2_S production and bladder band relaxation (118). The excision of the urothelium and the use of CBS inhibitors both markedly enhanced carbachol-induced contractions in human bladder strips. Additionally, activation of β3 receptors in the human urothelium has been shown to stimulate H_2_S production and promote bladder relaxation. CSE, but not the CBS inhibitor, was found to increase BRL37344-induced relaxation, a response that was abolished following epithelial removal (117). These findings suggest that H_2_S may play a role in modulating bladder function as a neuromodulator.

In addition, H_2_S also causes relaxation of bladder smooth muscle by activating ion channels. It is now generally confirmed that H_2_S induces bladder relaxation by activating K_ATP_ channels and mechanisms that desensitize Ca^2+^ (25, 114, 119). On the contrary, the BKCa channel, another frequently researched ion channel, has sparked controversy. Fernandes et al. (115) demonstrated that GYY4137 directly inhibited BKCa channels activity and decreased BK channels open probability in guinea pig bladder smooth muscle, leading to an increase in spontaneous phasic and neurally evoked contractions. Conversely, inhibiting BKCa channels in pig bladder neck smooth muscle has been found to diminish the relaxant effects of rolipram, a PDE4 inhibitor, with the observed impact of rolipram being partially attributed to H_2_S released from neurons. Essentially, the relaxation of pig bladder neck smooth muscle may be facilitated by H_2_S activation of BKCa channels (119). The conflicting outcomes of the two studies underscore the necessity for additional research, particularly considering the absence of urothelium in the bladder tissues utilized. Factors such as species specificity and the quality of experimental reagents must also be taken into account. Recent advancements in the field have identified PDE inhibitors as promising therapeutic options for the management of LUTS and ED (120). Numerous investigations have demonstrated the involvement of H_2_S in the mechanism of action of these drugs, with rolipram exhibiting significantly greater efficacy compared to sildenafil (119, 121, 122).

In conclusion, the relaxation effect of H_2_S on bladder smooth muscle is beneficial to relieve LUTS inclusive of overactive bladder, thereby removing the primary cause of related ED and improving the life quality of patients in many ways.

## 5 Conclusion

H_2_S, the third gas signaling molecule after NO and CO, is involved in various pathophysiological processes in the male reproductive system. In particular, it promotes penile erectile function, protects testicular function, inhibits the progression of PCa, regulates the spontaneous contraction of VD, and maintains the hypomotility of sperm in the cauda epididymis before ejaculation. A complex mechanism by which H_2_S relaxes smooth muscles in the penis may involve activating BKCa and Kv channels, inhibiting RhoA/ROCK signaling pathways, and raising cGMP levels to bypass NO pathway. Recently, selective β3 agonists such as mirabegron (75) and natural extracts such as RVT (79) and STS (77) have been shown to improve ED, with both classes of compounds being at least partly dependent on H_2_S. Besides, the relaxation of bladder smooth muscle by H_2_S has been shown to improve LUTS and subsequent ED. Consequently, the multifaceted therapeutic benefits of H_2_S offer potential for reducing medication dosages and enhancing the quality of life for patients with both conditions. Therefore, H_2_S is a promising target for the clinical treatment of male reproductive system diseases.

**Figure 3:**
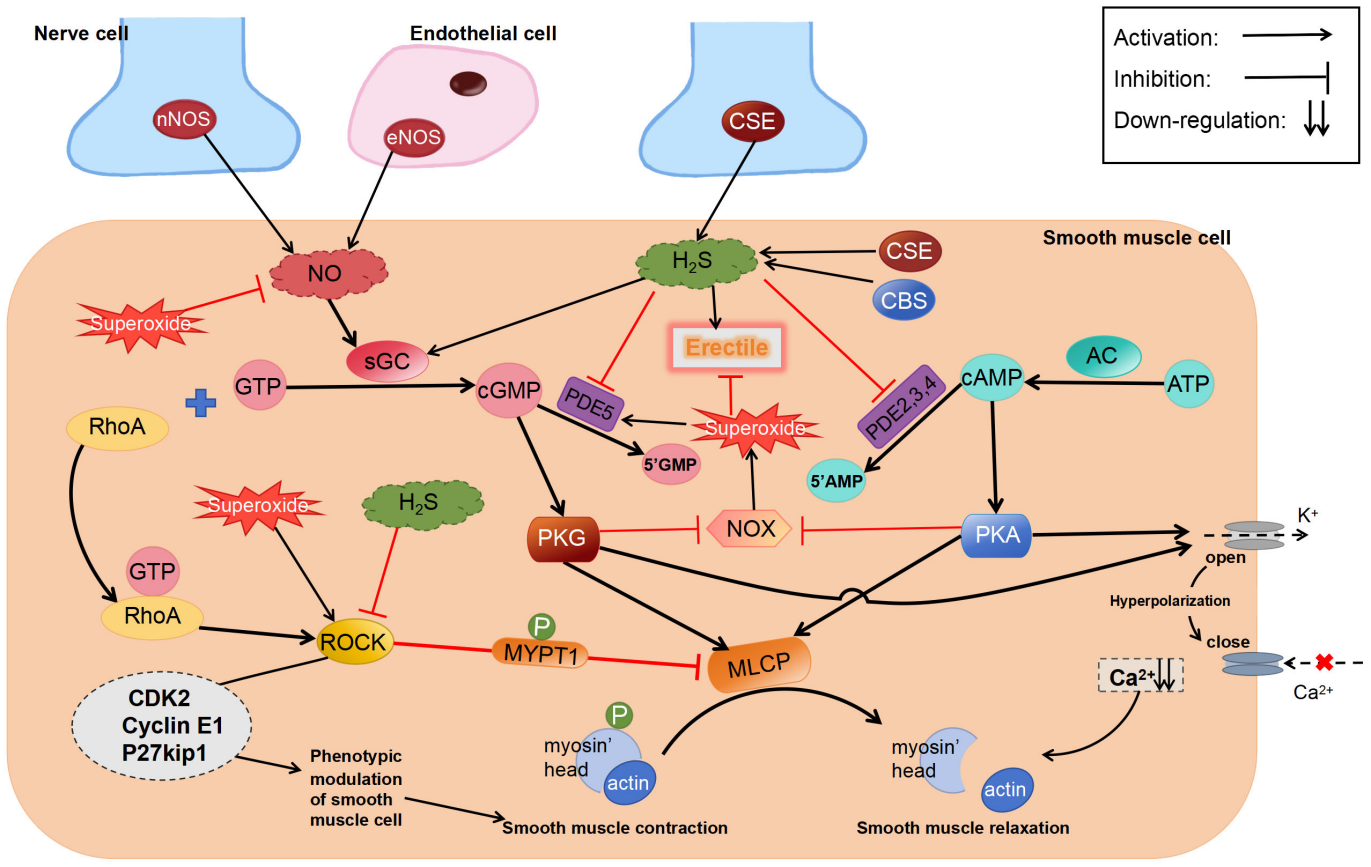
Regulatory effects of H_2_S on erectile dysfunction. ROCK, Rho-kinase; sGC, soluble guanylate cyclase; MYPT1, myosin phosphatase-targeting subunit 1; cGMP, cyclic guanosine monophosphate.

The process of H_2_S promoting penile erection may involve a variety of mechanisms, however, a unanimous final conclusion has not yet been reached. There is a very interesting theory in the literature on ACS6 treatment of ED (78) and this theory (**Figure 3**) is as follows: Superoxides derived from NADPH oxidase (NOX) react with NO to form active nitrogen in CCSMCs and arterial SMCs, thereby reducing the bioavailability of NO and attenuating penile erection (123–126). NO inhibits the activity and expression of NOX via the cGMP/PKG pathway (127–129). However, in patients with ED with diseases that impair endothelial function, such as diabetes, decreased NO levels lead to a reduction in cGMP expression, which in turn attenuates the inhibitory effects of NO on NOX and increases the production of superoxides, eventually forming a vicious circle. These superoxides upregulate PDE5 expression, resulting in the increased metabolism of cGMP. Similar to NO, H_2_S inhibits the activity and expression of NOX via the cAMP/PKA pathway (130). NaHS can activate PKG dependent on cGMP (131). In addition, the superoxides derived from NOX can activate ROCK (132).

Although the abovementioned theory has not been comprehensively investigated, we speculate that “inhibition of superoxide production” is a more reasonable explanation for the contradictory results of existing studies. As well as activating PKA and PKG, H_2_S inhibits superoxide generation by regulating Nrf2 and downstream anti-oxidative stress proteins, such as SOD, NQO1, and HO-1, via S-sulfhydration of Keap1 (133). In addition to activating K_ATP_ (134) and BKCa (55) channels, H_2_S-mediated S-sulfhydration effect reduces PDE5A dimerization (135). Therefore, irrespective of the type of pathway inhibitors used, they can only partially prevent the relaxation induced by H_2_S.

Although exogenous H_2_S has been shown to alleviate ED, further investigation into the mechanisms through which H_2_S promotes erectile function may guide the development of targeted drugs. Moreover, the role of endogenous H_2_S in promoting penile erection remains uncertain. Ghasemi et al. (26) found that PAG increased NANC relaxation in rat CC, which may be attributed to the inhibition of NOS activity by H_2_S or the direct chemical reaction of H_2_S with endogenous nitrogen oxides, such as NO, NO^+^, and HNO. Based on this hypothesis, the authors divided rat CC strips precontracted with PE into three groups, which were treated with S-nitrosoglutathione, SNP, and Angeli’s salt (three nitrogen oxide donors), respectively. Subsequently, each group was treated with NaHS at doses of 30 nM, 300 nM, or 30 μM. The results showed that all three nitrogen oxide donors induced the relaxation of rat CC tissues in a concentration-dependent manner; however, treatment with NaHS remarkably inhibited the effects of Angeli’s salt. These findings indicate that NO participates in the regulation of erectile function mainly in the form of HNO and is inhibited by H_2_S. Some studies have suggested that the physiological concentration of H_2_S is at the nanomolar level (136). Ghasemi et al. showed that CC relaxation was observed only after treatment with NaHS. Therefore, endogenous H_2_S may inhibit nitrogenous relaxation in CC. However, to date, limited studies have focused on this topic, and further research is warranted to determine the regulatory effects of endogenous H_2_S on CC relaxation.

In addition to its effects on the penis, H_2_S has diverse effects on other male reproductive organs, which warrant an in-depth investigation. The expression of CBS is not considerably altered in hormone-dependent and drug-resistant human PCa tissues (30). Even if it is increased after neuroendocrine differentiation of PCa cells, it does not appear to be involved in the effects of H_2_S (47). However, overexpression of CBS in the testis can alleviate spermatogenesis disorders (51), and CBS/H_2_S can regulate testosterone synthesis (100) Therefore, the use of CBS as a therapeutic target for restoring testicular function may minimize the risk of side effects on the prostate. Given that successful ejaculation requires smooth muscle contraction in the VD, seminal vesicle, and prostate (137), drugs targeting H_2_S in the VD may be used to treat premature ejaculation. However, the effects of H_2_S on the VD suggest that the half-life of H_2_S should be controlled for a certain period to prevent ejaculation disorders and maintain normal sexual function and effective sexual behavior in men. In conclusion, H_2_S possesses great potential in the treatment of male reproductive system diseases.

## Author contributions

YS: Writing – original draft. CM: Writing – original draft. QZ: Writing – original draft. RZ: Writing – original draft. DJ: Writing – review & editing. XS: Writing – review & editing.
